# School Mindfulness-Based Interventions for Youth, and Considerations for Anxiety, Depression, and a Positive School Climate—A Systematic Literature Review

**DOI:** 10.3390/children10050861

**Published:** 2023-05-11

**Authors:** Jessica Monsillion, Rafika Zebdi, Lucia Romo-Desprez

**Affiliations:** 1 UR 4430 Clipsyd, Department of Psychology, Université Paris Nanterre, 200, Avenue de la République, 92001 Nanterre, France; 2 Hopital Raymond-Poincaré (APHP), Inserm CESP 1018 UPS, 104 Bvd Raymond Poincaré, 92380 Garches, France

**Keywords:** anxiety, child, depression, mindfulness-based interventions (MBIs), school climate

## Abstract

Mindfulness-based interventions (MBIs) are growing in popularity, with research concerning their efficacy with youth populations. Following a preliminary analysis of the existing literature, and given the positive effects of such programs, we felt it relevant to assess whether research has considered the implications for MBIs on children and adolescents, with regard to depression, anxiety, and school climate. Objectives: We aim to estimate the effect of MBIs as innovative interventions addressing youths in school settings, with special consideration for anxiety, depression, and school climate outcomes. Method: This review investigates the existing literature in the field of mindfulness, using quasi-experimental and randomized control trial (RCT) models, targeted at youth (5–18 years) in school settings. A search was carried out in four databases—WebofScience, Google Scholar, PubMed, and PsycARTICLES. This resulted in 39 articles, which were sorted based on predetermined inclusion criteria; 12 articles qualified. Results: The results show discrepancies in terms of methodological and implementation variables, types of interventions, instructor trainings, assessment measures, and choice of practices and exercises, which make the effects of existing school MBIs difficult to compare. Consistencies were observed in emotional and behavioral regulation, prosocial behaviors, and reducing stress and anxiety in students. The results of this systematic review also suggest that MBIs could be potential mediators in improving student well-being and environmental factors, such as school and class climates. Specifically, children’s sense of safety and community can be improved by an improved quality of relationships between students, their peers, and teachers. Future research should consider incorporating school climate perspectives, such as implementing whole-school MBI approaches and using replicable and comparable study designs and methods, whilst considering the capacities and limitations of the academic and institutional context.

## 1. Introduction

This study explores the effect of mindfulness-based interventions (MBIs) on school climate and students from preschool levels to high school. Mindfulness is described as the act of deliberately focusing and maintaining awareness on the present moment, nonjudgmentally, as it unfolds, moment by moment, without emotional reactivity, denial or mental rigidity regarding what is occurring [1,2]. Originally, a Buddhist meditative practice, mindfulness has since been secularized for clinical and medical applications. Indeed, mindfulness has been developed and adapted for therapeutic purposes in behavioral practices in a variety of contexts, both in practices that exclude meditations, such as in acceptance and commitment therapy [3] and dialectical behavioral therapy [4] and in specific meditation trainings, such as mindfulness-based stress reduction [1] and mindfulness-based cognitive therapy [5], or in brief mindfulness-based interventions (MBIs) [6,7,8]. The operational definition of mindfulness is: (1) Attention self-regulation, directed and focused on immediate experiences, allowing increased awareness to internal and external events in the present moment; and (2) Awareness, curiosity, and acceptance towards experiences in the present moment [9]. MBIs aim to teach and cultivate such skills to help cope with difficult and stressful situations, throughout structured practices focused on intentional awareness and acceptance of bodily sensations, emotions, and thoughts in the present moment. In recent decades, MBIs have grown exponentially as psychotherapeutic protocols in preventing emotional disorders and in dealing with chronic pain. Research has reported that the practice of mindfulness supports people in changing the nature of their relationships with such experiences, and in the long run, has positive effects on diverse outcomes such as cognitive abilities, stress levels, and prosocial behaviors [10,11,12]. Albeit less conclusive, recent systematic reviews and meta-analyses demonstrate that MBIs are associated with a decrease in anxiety and depression symptoms [13] and with positive effects on emotional regulation strategies [14]. Indeed, there has been a concurrence of evidence supporting that participating in MBIs leads to greater satisfaction in life and increases psychological health in both clinical and non-clinical adult populations [15].

Given these outcomes on adult populations, an increasing number of MBIs has been adapted for children and adolescents [8,16,17] as early prevention and intervention strategies aiming to minimize mental health risks. The Learning to BREATHE (L2B) program [16] is an MBI aiming to teach social–emotional skills to adolescents. Its effectiveness was assessed in a public high school (n = 216 students); post-intervention participants reported higher abilities of emotional regulation (emotional awareness, access to emotional regulation strategies, and emotional clarity) and lower levels of perceived stress and psychosomatic complaints. Mindfulness-based cognitive therapy for children (MBCT-C), developed by Semple and Lee (2014), is a 12-session mindfulness-based program adapted for children aged 9 to 12 suffering from anxiety and other internalized symptoms. Sessions were shortened and made more repetitive to better fit the cognitive abilities of children. Mindfulness skills are taught through experiential learning activities such as sensory-based practices, drawing, writing, and visualizations. This program proved helpful in reducing internalizing and externalizing symptoms in children who reported clinically high levels of anxiety at pre-test, resulting in fewer attention problems, which were maintained three months following the intervention [17].

Provided that MBIs are adaptable and beneficial to youth populations with regard to anxiety, depression, and emotional regulation, research suggests that protocols are further investigated in schools and applied in educational settings [8,18]. Moreover, the literature shows that school-based health and well-being interventions seem to effectively reduce mental health issues for students [19]. Therefore, the question of whether mindfulness practices are suitable as universal preventive interventions in educational settings has been raised, and a number of MBIs have been implemented in schools in order to promote health and improve accessibility to mental health services [20]. Most school-based MBIs measure a variety of outcomes, and therefore, programs tend to differ in content. Nevertheless, preliminary evidence suggests that school-based MBIs can benefit the well-being of students and teachers in the classroom [21], lead to higher academic success by enhancing cognitive performances and executive functions, and have positive effects on socio-emotional processes [22]. Research also suggests MBIs’ effectiveness on emotional and behavioral regulation in youth [23], facilitating prosocial behaviors and mental health, such as decreasing symptoms of anxiety and depression [24].

Despite the existence of many systematic literature reviews addressing MBIs in school settings, few of these consider the implications of such interventions on school climate. This, however, is an important consideration as a positive school climate acts in favor of students’ psychological, physical, and cognitive well-being [25]. There is no universally accepted definition of school climate or its specific features; however, the construct of a positive school climate has been recognized to enhance student achievement and reduce problematic behaviors across definitions [25]. Furthermore, a positive school climate has been shown to encourage resilience in students, while negative school climates correlate to risk factors for students and faculty [26]. Wang and Degol offer a multidimensional definition and construct of school climate (2016). Authors define four categories they consider to be at the root of a positive school climate: (1) Safety (sense of physical and emotional security provided by the school, discipline, and order); (2) Community (quality of social interactions and relationships at school); (3) Academic (quality of the curriculums, teacher training, instructions, and professional development); and (4) Institutional environment (structural organization, adequacy, and availability of resources). According to this definition, a positive school climate is made possible by the quality of the four factors that will shape students’ emotional, behavioral, cognitive, and social development. Many of the underlying elements that make up these categories, such as those pertaining to safety and community, could be targeted for improvement through the implementation of mindfulness practices in the school context. Therefore, in this article, we have directed our focus on the effects of school MBIs on the shaping of students’ sense of safety, community, and well-being with regard to academia. Moreover, school climate and classroom climate are most commonly studied as two different concepts and refer to two different nested systems. However, Evans et al. (2009) [27] determined three common component of a positive classroom climate: (1) Academic (quality of curricular elements); (2) Management (referring to order and disciplinary style); and (3) Emotional well-being, social interactions, and relationships within the classroom. These overlap with three of Wang and Degal’s components of positive school climate. These components suggest that when students perceive these factors as positive in class, emotional well-being (safety), interpersonal relations (community), instructional and classroom management (academic), improvements in academic achievement and overall success are witnessed [28,29]. Therefore, this review includes studies that have also considered classroom climate with regard to safety, community, and academic environment.

Given that mental health issues continue to grow in school-aged populations, it is especially important to find ways to curb this issue, as we continue to see a link between socio-emotional development and academic performance [30,31]. Mindfulness practices have been shown to positively influence factors, such as stress and personal well-being in school children, as they promote awareness and self-regulation, more specifically, they increase emotion and attention regulation by developing cognitive control, sensory awareness, and acceptance of momentary thoughts and feelings [29]. Additionally, some programs have indirectly had the potential to act on specific factors in the equation for school climate—such as the Mindfulness-Based Wellness Education Program, which effectively targets burnout and stress in teachers [32]—and found a significant increase in teacher well-being, which positively reflected on teacher–student relationships. Another example is the Learning how to BREATHE program [16], which teaches high school students mindfulness techniques in order to improve school climate. Similarly, Strongkids is a program developed specifically for teaching children mindfulness in a school environment [33]. These MBIs have shown their adaptability and feasibility in an educational setting; however, these programs are rarely implemented in elementary and middle schools, and the existing studies do not address their impact on the concept of school climate—especially throughout Europe. Nonetheless, various positive outcomes from the implementation of school MBIs have been witnessed and seem to overlap with key factors for positive school climate as defined by Wang and Degol [25], such as academic success [34,35] overlaps with the improvement of cognitive performances and executive functions [36], and social and emotional well-being with a decrease in psychological symptoms (stress, anxiety, and depression) and problematic behaviors [23,24]. Hence, it is important for research to consider the implications of mindfulness not only on individuals’ well-being, but also on well-being with regard to school climate overall.

Accordingly, the present article seeks to elucidate the effectiveness of school MBIs in preschool, elementary, secondary, and high school students (between 5 and 18 years old), considering mental health and school climate perspectives. We hypothesized that consistencies would be found across interventions regarding the effects of school MBIs on children and adolescents (decrease in stress, anxiety, and depression) and on improving school and/or class climate. Therefore, the purpose of this study is to review the main findings in this novel field of research by highlighting consistencies and inconsistencies of school MBIs, assessment methods, and the main effects of school MBIs on improving children’s and adolescents’ mental health and well-being, and the quality of school climate. To meet this objective, a systematic literature review was conducted. The general objective was to describe and examine empirical research with experimental designs that have implemented and assessed the effects of school-based MBIs as innovative and accessible interventions to strengthen school climate and reduce child and adolescent mental health problems.

## 2. Materials and Methods

### 2.1. Search Strategy

We conducted a systematic search of 4 databases from June to November, 2022. This systematic search was carried out using the guidelines proposed in the preferred reporting items for systematic review and meta-analysis (PRISMA) [37], as these guidelines offer a complete and comprehensive overview for conducting an efficient review. The search of articles was limited to published studies between 2010 and 2022, as a prior screening of the literature on school MBIs revealed that this domain of research is a relatively new field of application. We used the following databases: Google Scholar, Web of Science, PsycARTICLES, and PubMed, using the following search terms: “mindfulness-based interventions”, AND child*, AND anxiety, AND depression, AND school climate*.

### 2.2. Eligibility Criteria

The following inclusion criteria were determined prior to the search: (1) Studies must focus on the use of mindfulness-based interventions (MBIs); (2) Participant ages should range from 5 to 18 years old, focusing on primary/elementary to high school students (even if mindfulness practices have shown their positive effects on younger populations, we consider school climate and student academic well-being at pre-school levels to be significantly different from primary and high school climate due to early childhood developmental stages and academic goals); (3) Interventions must have taken place in a school setting; (4) Students must not have been chosen based on specific qualities (i.e., learning difficulties and mental health disorders), as the intended focus is on potential universal implementation; (5) Studies with empirical results; (6) Articles must have been peer-reviewed. As the literature investigating a direct relationship between school climate and mindfulness is minimal, we searched for studies that have taken school climate into consideration in any stage of their studies, so we added our final inclusion criterion; (7) Articles must consider school climate and/or class climate. Additionally, articles were excluded if: (1) Participants were younger or older than the age range of 5 to 18 years old; (2) Studies were conducted at a pre-school or university level; (3) Studies tested interventions consisting of similar practices or other contemplative practices (i.e., yoga and Tai Chi) without an explicit mention of mindfulness meditation; (4) Participants were selected based on certain features (psychiatric diagnosis, special aid classes, etc.); (5) If the source was a review, grey literature or a commentary. All search results were included in our dataset before screening regardless of relevance. Articles were gradually excluded from further consideration based on title and abstract relevance. The search process was carried out by the author of the article and the research assistant. Each reviewer was assigned two databases, for which they then reviewed each search result. However, both reviewers were able to review one another’s work, as they made use of a communal findings dataset. In this way, the author had the ability to supervise whether the inclusion/exclusion criteria had been thoroughly examined for each individual search result. Had any doubts or inconsistencies in the inclusion/exclusion criteria arisen, a set process of verification would have been conducted by a third party (third author of the study) to resolve doubt. However, this was not the case and no inconsistencies arose. See Table 1 for included studies characteristics.

### 2.3. Data Extraction and Synthesis

Data extraction was based on the PICOS (population, intervention, comparator, outcomes, and study) methodology. A Prisma checklist and flow diagram were used in the data search, selection, and inclusion and exclusion process. Considering the small body of literature, we included studies that met our defined criteria (experimental RCT design or quasi-experimental study designs with pre- and post-test results, MBI, school intervention, children 5 to 18 years old, general population, anxiety/depression, and school and class climates) regardless of their level of evidence and risk of bias, but interpreted these findings with caution. We also concluded that given the few existing studies, conducting an empirical synthesis of the findings or meta-analysis was not relevant at the time. 

### 2.4. Quality Assessment

We referred to the National Institute of Health Quality Assessment of Controlled Intervention Studies for quality assessment (https://www.nhlbi.nih.gov/health-topics/study-quality-assessment-tools; URL (accessed on 4 July 2022)) (Table 2). Yet, as mentioned previously, we included studies meeting our inclusion criteria regardless of their level of evidence and risk of bias but interpreted these findings in the context of possible bias.

## 3. Results

### 3.1. Study Selection and Characteristics

**Figure 1:**
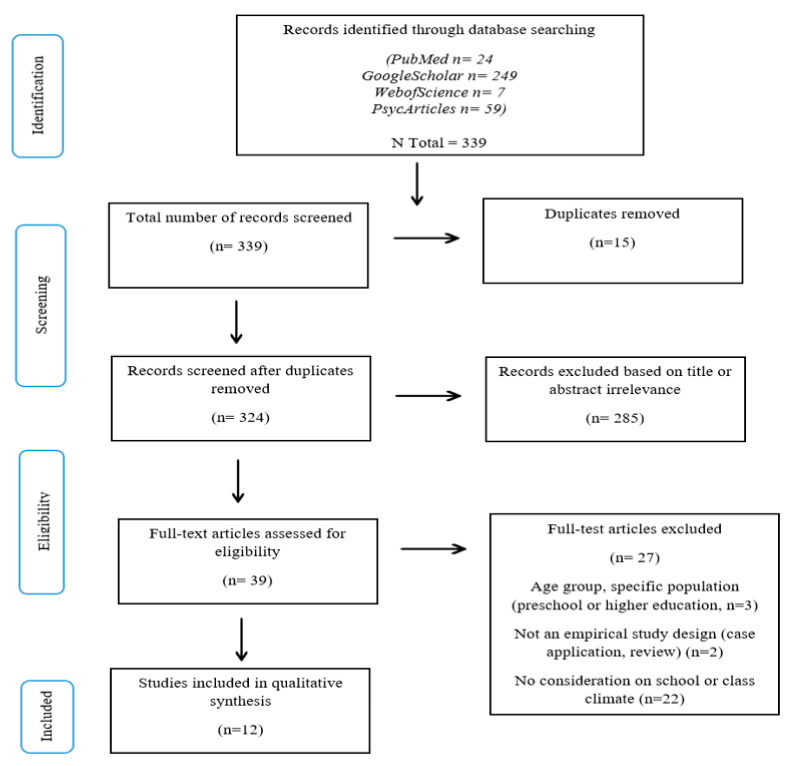
PRISMA flow diagram.

Initially, the search revealed 339 results from PubMed (24), GoogleScholar (249), WebofScience (7), and PsycArticles (59). These references were screened for duplicates, and 15 were removed, resulting in a total of 324 references. The relevance of each article was determined based on the article name and abstract—285 were excluded based on title and abstract irrelevance. We then retrieved full-text articles for the remaining 39 references, once again excluding results that did not meet the predetermined inclusion/exclusion criteria. We screened each of these articles and included studies by: (1) Type of study: studies with empirical results (excluding: reviews, grey literature, reports, commentaries, meta-analysis or systematic reviews); (2) Population: general population, children aged 5 to 18 years old, students at an elementary and/or high school level (excluding: university level); (3) Intervention: school-based mindfulness intervention (MBI); (4) School and classroom climate: considerations for improving at least one of the following: sense of safety, community, and academic and institutional environment. Twenty-seven studies were excluded because they did not meet the above-mentioned requirements. Thus, 12 studies fulfilled all the eligibility criteria and were included in the qualitative synthesis. See Figure 1 for the PRISMA flow diagram [38] depicting these findings. Of these twelve studies, five took place in the United States of America, three in Spain, one in Canada, one in the Netherlands, one in the United Kingdom (England), and one in Brazil. The studies were published between 2012 and 2020. Nine of these studies were controlled intervention studies, five of which were randomized [39,40,41,42,43]. Only one included study used active control groups [41] and eight used inactive “wait-list” control groups [39,40,42,43,44,45,46,47]. Three one-group pre-experimental studies applied a pre-test–post-test study design with no control group [48,49,50], one of which is also a longitudinal study [49].

Table 1 presents the characteristics of these twelve studies, and Table 2 presents an evaluation for risk of bias using the National Institutes of Health’s study quality assessment tools for controlled intervention studies and the quality assessment tool for before–after (pre- and post-intervention) studies with no control group.

**Table 1:** Included studies’ characteristics.

N°	Study	Country	Design	Population/Sample (N)	Intervention	Consideration for School & Class Climate
1	Waldemar et al., (2016) [47]	Brazil	Quasi-experimental design (pre/post-test measures) with inactive matched control group: MBI vs. control group Non-randomized	5th grade (mean age 11.1) Elementary school N = 120	M-SEL: Mindfulness—Social–Emotional Learning	Community: Yes Safety: Yes Academic: No Institution: No
2	Van de Weijer-Bergsma et al., (2012) [39]	Netherlands	RCT: MBI vs. wait list control group	Aged 8 to 12 years (mean age 9.92) Elementary school N = 199	MindfulKids	Community: Yes Safety: Yes Academic: No Institution: Yes
3	Kielty et al., (2017) [49]	USA	One-group pre- and post-test pre-experimental design No control groups Longitudinal	Third grade (age not specified) elementary school N = 45	Curricula designed by authors, based on Mindful Schools and MindUp	Community: Yes Safety: Yes Academic: No Institution: No
4	Schonert-Reichl et al., (2015) [41]	Canada	RCT with active control group: MBI vs. control group (SEL and Mind vs. Business as usual social responsibility program)	Aged 9 to 11 (mean age 11.16) Elementary school N = 99	Social and Emotional Learning (SEL) combined with mindfulness based on MindUp intervention curricula	Community: Yes Safety: Yes Academic: Yes Institution: No
5	Bradley et al., (2018) [50]	USA	One-group pre- and post-test pre-experimental design No control group	Mean age 9.3 Elementary school N1 = 49 Teachers; N2 = 507 children	The Four Pillars of Well-Being	Community: Yes Safety: Yes Academic: No Institution: No
6	Parker et al., (2014) [40]	USA	RCT: MBI vs. wait list control group	Aged 9 to 11 years (mean age 10.09) Elementary school N = 111	MasterMind	Community: Yes Safety: Yes Academic: Yes Institution: No
7	Wisner, Betsy (2014) [48]	USA	Exploratory study: pre- and post-test and mixed-method approach (concept mapping) No control group	High school grades 10, 11, 12 (mean age: 17.89) N = 35	Mindfulness meditation (MM)	Community: Yes Safety: Yes Academic: No Institution: Yes
8	Kuyken et al., (2022) [42]	UK	Study protocol for cluster randomized controlled parallel group trial (inactive)	Aged 11 to 16 years old (students) N = 672 (teachers) N Schools = 85 (approx. 1000 students)	School-based mindfulness training (SBMT)	Community: Yes Safety: Yes Academic: Yes Institution: Yes
9	Lombas et al., (2019) [44]	Spain	Quasi-experimental design (pre/post-test measures) with controlled group (inactive)	(Mean age: 13.6 years, Grades 7, 8, 9, 10) N = 524	Happy Classrooms Program (HCP)	Community: Yes Safety: Yes Academic: Yes Institution: No
10	Suárez-García et al., (2020) [46]	Spain	Quasi-experimental switching replications design (pre/post-test measures) with controlled group (inactive)	Aged between 7 and 10, 3rd year primary (mean age: 8.08) N = 73 (students) N = 5 (teachers)	Mindkeys training	Community: Yes Safety: Yes Academic: Yes Institution: No
11	Lauren Meyer & Katie Eklund (2020) [45]	USA	Quasi-experimental design (pre/post-test measures) with controlled group (wait list)	4th grade and 5th grade elementary (mean age: 9.3) Students N = 296 Teachers N = 14	Mindful Moments Intervention	Community: Yes Safety: Yes Academic: Yes Institution: No
12	Moreno-Gómez, Luna, & Cejudo, (2020) [43]	Spain	Quasi-experimental design (pre/post-test measures) with controlled group (inactive)	Aged 5 to 6 years (mean age: 5.69) N = 114	Mindkinder	Community: Yes Safety: Yes Academic: Yes Institution: No

**Table 2:** Quality assessments according to National Institutes of Health’s study criteria.

Quality Assessment Tools for Controlled Intervention Studies									
Study	Randomization Method	Treatment Allocation Concealed	Blinding of Patients and Providers	Blind Assessors	No Base-Line Group Differ-rence	Drop-Out Rate >20%	Drop-Out Rate between Groups >15%	Treatment Protocol Adherence	Other Treatment Avoided or Similar
Van de Weijer-Bergsma et al. (2012) [39]	Good	Fair	Poor	NR	Good	Good	Good	Good	Good
Schonert-Reichl et al. (2015) [41]	Good	Fair	Poor	Good	Good	Good	Good	Good	Good
Parker et al. (2016) [40]	Good	Fair	Poor	NR	Good	Good	Good	Good	Good
Waldemar et al. (2016) [47]	NA	NA	Poor	NR	Good	Good	Good	Good	Good
Suárez-García et al. (2020) [46]	NA	NA	NR	NR	Good	Good	Good	Good	Good
Meyer & Eklund (2020) [45]	NA	NA	NR	NR	Good	Good	Good	Good	Good
Moreno et al. (2020) [43]	Good	Fair	NR	NR	Good	Good	Good	Good	Good
Lombas et al. (2019) [44]	NA	NA	Poor	NA	NR	NR	NR	Good	Good
Kuyken et al. (2022) [42]	Good	Fair	Poor	NR	Fair	Good	Good	Good	Good
**Quality Assessment Tool for before–after (pre–post) studies**									
**Study**	**Study question**	**Eligibility criteria and population**	**Study participant representative of population of interest**	**1. Enrolment of all eligible participants** **2. Sample size**	**Inter-vention clearly described**	1. Outcome measure * 2. Blinding **	**Follow-up rate**	**1. Statistical analysis and** **2. Multiple outcome measures**	**Group-level interventions/individual level outcome efforts**
Wisner, Betsy (2014) [48]	Good	Good	Good	1. Fair 2. Poor	Good	1. Fair 2. NR	Poor	1. Good 2. Poor	Good
Kielty et al. (2017) [49]	Good	Good	Fair	1. Fair 2. Fair	Fair	1. Fair 2. NR	Poor	1. Good 2. Poor	Good
Bradley et al. (2018) [50]	Good	Good	Good	1. Fair 2. Good	Good	1. Fair 2. NR	Poor	1. Good 2. Fair	Good

NA: not applicable; NR: not reported; * Outcome measures clearly described, valid, reliable; ** Blinding of outcome assessors.

### 3.2. Quality of Included Studies

MBIs were delivered as part of the academic curriculum and set in the classrooms in all of the twelve included studies. In studies with randomized designs (n = 5), the schools were first contacted and if the teachers were interested, schools and classes were randomly allocated to either a mindfulness or control group. In one randomized study, classes were matched to school and grade when two parallel grades participated within one school [39]. They were then randomly assigned to an immediate-intervention group or a waitlist control group. Four studies using controlled comparison groups included samples that were not randomly selected or assigned [44,45,46,47]. In one of these pre-experimental (one-group pre-test–post-test design) studies, the experimental and control groups were designated by the schools to guarantee that the MBI could be delivered without adding requirements that would make its feasibility harder or disturb the school schedule [47]. Suárez-García et al. [46] applied a switching replication design splitting the targeted students into two groups by following the natural organization of the class groups. Three studies did not include control groups, and one of these studies applied a longitudinal approach by assessing a school MBI with third grade students from six different classrooms at a local elementary school over three years [49]. All studies evaluated the effects of MBIs at pre- and post-test (before and after the intervention). Four studies included baseline measures prior to the pre- and post-test data collection and reported follow-up measures [39,41,42,44]. Eight studies reported attrition data and specified reasons for participation withdrawal: participants moving away, school transferring, parental non-authorization, problems in the completion of questionnaires, and missing baseline data collection. Only one study specified taking attendance rate into account [39]. Overall, we observed low attrition rates, which varied between 1% [41] and 15.5% [39].

### 3.3. Participant Characteristics

Across these twelve studies, participant characteristics can be regrouped in three populations: types, students, and teachers. The children sample sizes ranged from 45 to 524. MBIs were delivered to elementary-school-aged children in third to sixth grade; mean ages ranged from 9.3 to 11.16. Middle-school-aged children are children in 7th to 9th grade, and high school students are those in 10th to 12th grade. Teachers were all elementary, middle school, and high school teachers. The sample sizes of teachers ranged from 3 to 672 (Table 1). In eight of the included studies, the teachers were trained to deliver the MBIs in class [40,41,42,43,44,45,46,50]. One MBI was delivered by trained mindfulness professionals, but included teachers during sessions who were asked to perform a five-minute exercise with the class on the remaining school days [39]. Two studies had the MBIs delivered by licensed therapists that had extensive training in the intervention and a personal mindfulness practice [48], and one had young psychologists training to be psychotherapists deliver the MBI program [47].

### 3.4. Outcome Measures

With regard to children’s and adolescent’s personal well-being outcomes, the included studies examined executive functions and related constructs, such as attention (n = 3) [40,41,46], emotional and behavioral regulation (n = 7) [39,40,41,43,46,47,48], and emotional problems (depression and anxiety) (n = 7) [39,40,41,43,44,47,50] (see Table 3). Two studies examined the risk for attention deficit and hyperactivity disorder (ADHD) [46,47]. One of the published reports investigated intentions to use substances (alcohol and tobacco) [40].

Some of the included studies examined social and environmental factors. Three studies examined prosocial behaviors and related constructs, such as empathy, theory of mind, social responsibility, and peer acceptance [39,41,44]. One study evaluated social–emotional factors, such as resilience and optimism [41]. Regarding environmental elements, six published reports investigated either psychosocial adjustments at school [39,41,42,44,45,49] or quality and satisfaction of life and needs [39,44,47]. Three studies investigated academic improvements [41,43,44]. The mindfulness student trait was measured in three of the included reports [41,44,49]. School MBI feasibility and program acceptability were studied in six studies [40,41,44,48,49,50]. Studies used a combination of direct student assessment measures and/or teacher-rated measures, often reporting outcomes from both sources in the published articles. One study used parent-rated assessment measures [39]. With regard to teacher samples, studies examined the effects of school MBIs on various teacher dimensions. Three articles assessed the trait mindfulness [42,45,50]. Three included published reports studied the effects on the environment (school and/or class climate) [39,42,45]. Two studied and assessed the broader category of psychological and environmental well-being, either in the scope of burnout-related factors, such as teacher sense of self-efficacy and job satisfaction [50], emotional factors such as stress, anxiety, depression, contentment/positive emotions, and self-compassion [42,50], or social aspects (relationships) [50].

#### 3.4.1. School-Based Mindfulness Interventions

Over all, the objectives in these studies were to test the feasibility of MBIs in a school context and to assess the effects of such programs on mental health (emotional problems, conduct problems, prosocial behaviors, and stress reduction), self-regulation, social–emotional competencies, and quality of life (teacher–student relationship and classroom and school climates) of students in preschool, elementary school, and high school. As can be seen in Table 4, eight studies tested MBIs that were theorized and developed based on previously existing mindfulness programs, such as MBSR and MBCT (MindUp, MindfulKids, MasterMind, Mindkeys, MM, SBMT, Mindkinder, and Mindful Moments). Two of the studied interventions combined mindfulness with a pre-existing school-based intervention program, Social and Emotional Learning (SEL). The most recent publication in this review developed a school MBI called “The Four Pillars” combining positive psychology, mindfulness, and social and emotional learning to enhance the understanding of personal well-being, self-awareness, and to maintain a positive classroom climate for teachers and students [50]. Manualized MBIs, such as M-SEL (Mindfulness–Social Emotional Learning), The Four Pillars, Mastermind, Happy Classrooms Program, Mindful Moments, were available but only two had an enduring presence of over five years. For these manualized programs, extra guidance material for implementation, assessment, and trainings are easily found on the referenced websites.

As shown in Table 4, seven MBIs were implemented by class teachers trained in delivering the mindfulness programs [40,41,42,43,44,45,50]. The other five were conducted by non-school professional mindfulness experts who were also involved as study authors [39,46,47,48,49]. In three studies, while trainers conducted the lessons, they also included teachers in the sessions [48]; teachers were also asked to deliver breathing exercises or other mindfulness activities daily, outside the sessions [39,46]. One MBI was used in two studies but implemented differently: one study delivered the M-SEL program by trained teachers (Waldemar et al., 2016) and one used professional trainers [41].

The interventions’ implementation periods and intensities, varied from three weeks to a year. Sessions were sometimes split into several shorter sessions a week, their duration ranging from 4 min to one hour (see Table 4). The shortest program lasted 3 weeks, with one ‘booster session’ in the two following years for a total of 5 sessions [49]. The longest intervention had 12 sessions spanning 28 weeks [39]. All MBI programs structure and facilitate sessions on different core mindfulness components (mindfulness and awareness of breath, body, senses, thoughts, emotions, orientation of attention, and empathy), and include psychoeducation on mindfulness, thoughts, emotions, behaviors, daily and home practices, and group discussions (see Table 4). Five studies added teachings of either social emotional learning or other holistic approaches, such as CASEL skills (collaborative for academic, social, and emotional learning), visualization exercises, mandala exercises, cultivating kindness and gratitude, character strengths and well-being practices (appreciation of beauty, gratitude, hope, humor, and spirituality) (see Table 4). In regard to positive school and class climates, all programs contain components that facilitate the effects on the safety and community factors (n = 12), seven programs on academic factors, and three on institutional factors (see Table 4).

#### 3.4.2. Stress & Anxiety

Parker et al. [40] observed changes in parent-rated anxiety scores at post-intervention. Van de Weijer-Bregsma et al. [39] also used parent-reported data to assess anxiety, using a questionnaire that considered five scales: panic disorder, social phobia, generalized anxiety disorder, obsessive compulsive disorder, and separation anxiety disorder. The findings of both Parker et al. [40] and Van de Weijer-Bregsma et al. [39] indicated significant decreases in anxiety scores at post-test. Teacher-rated anxiety decreased significantly in girls from the pre-test to the post-test when compared to the control group [40], although Van de Weijer-Bergsma et al. [39] found no such gender difference when analyzing parent-reported data. One study included the assessment of physiological stress via salivary cortisol levels three times within one day, relative to awakening, at both the pre-test and post-test but no significant differences were found [41]. However, students having participated in the MBI MindUP had significantly higher cortisol secretion at morning arrival at post-test than control group children. Bradley et al. [50] assessing teacher burn-out, anxiety, and stress found improvements in self-compassion, teaching efficacy, and feelings of contentment, which correlated positively with subjective well-being, self-compassion, and negatively with stress. These improvements had positive effects on students’ self-reported moods, which shifted into positive lower arousal states at post-intervention. 

#### 3.4.3. Depression

One study observed a decrease in depressive symptoms and significant improvements in optimism and perspective taking at post-intervention [41]. Waldemar et al.’s results at post-intervention showed significant improvements, which were self-reported by children who participated in the school MBI, in comparison to the control group [47]. Another study assessed rumination as a predictor/risk factor for depression and students’ subjective feeling of happiness but the results revealed that the intervention had no effects, from pre-test to post-test to follow-up [39]. However, the study found a small but significant decrease in rumination in the experimental condition, as reported in the self-reported data. They found rumination scores to correlate with other outcome effects; higher levels of rumination correlated to a greater decline in analyzing emotions when compared to participants with lower levels of rumination. Additionally, participants with lower scores for rumination gained greater attention to the emotions of others, as well as greater bodily awareness. Finally, children with lower rumination scores were found to show greater initial levels of aggression. These scores decreased upon completion of the intervention. Furthermore, Lombas et al. revealed that their school MBI improved several indexes of psychological well-being (relative to self-esteem and satisfaction with life, seen as a risk factor for depression) pointing to the conclusion that school mindfulness interventions could reduce depressive symptoms, such as perceived stress, while they increased empathy and life satisfaction [44]. However, regarding depressive symptoms, perceived stress, and amotivation, the intervention proved to have positive effects only when levels of trait mindfulness were high or medium at pre-intervention. Finally, authors point to the positive effect of MBIs on self-esteem.

#### 3.4.4. Emotional and Behavioral Regulation

Overall, we found that school MBIs positively impact emotional and behavioral problems by observing declines in stress, rumination, anxiety, depressive symptoms, and externalizing behaviors.

Emotional awareness: three studies assessed emotional awareness. One study used the Mood Meter [102], an emotion-plotting tool in the form of a grid used to visually represent the full spectrum of one’s emotional state [50]. Reports are made in terms of two dimensions “pleasantness” and “energy”, on a scale ranging from −5 (extremely unpleasant/low energy) to +5 (extremely pleasant/high energy). Mood and emotion word reports changes were significant between the two time points. Participants’ understanding of emotions, or emotional granularity [103], also increased. Authors observed a significant 12% increase in unique emotion words provided at post-intervention. Van de Weijer-Bergsma et al. applied the Emotion Awareness Questionnaire Revised [95], which assess children’s emotional functioning [39]. Emotional awareness, although the effect sizes were small, was positively impacted by the intervention as verbal sharing of emotions, not hiding emotions, sense of coherence, and bodily awareness of emotions increased significantly at post-test [39]. Results also suggest links between levels of rumination and attending to others’ emotions, as children with medium or low levels of rumination tended more to others’ emotions at post-intervention. Lombas et al. explored the effects of the MBI on emotional intelligence through the constructs of emotional attention, clarity, and repair [44]. They found that mindfulness mediated positive effects on emotional attention, but not on emotional clarity or emotional repair.

Emotional and behavioral control: six studies looked at the effects of school MBIs on emotional and behavioral control [40,41,43,46,47,48]. Waldemar et al. found that children who took part in the school MBI showed significant improvements in emotional control in contrast to the control condition (social responsibility program group) at post-intervention [47]. Another study used the Resilience Inventory Subscale [70] to assess emotional control [41]. It consists of five items assessing the degree to which the respondent feels he or she has some control over his or her emotional reactivity and emotional displays. Results at follow-up show that children having participated in the MBI had significant improvements in emotional control [41]. Parker et al. referred to self-regulation and self-control (encompassing the ability to modulate thoughts, behaviors, and emotions) and used the teacher-rated Self-Control Rating Scale [40,54]. At post-test, they found that teachers rated better self-control in boys having participated in the MBI compared to the control condition. In addition, Suárez-García et al. studied the effects of the MBI on self-control deficits using the “hyperactivity–impulsivity” subscale from the primary teachers’ version of SENA [46,57]. The results indicated that the most significant improvement from the intervention was the reduction in deficits in self-control. However, at follow-up, rather than continuing to improve, it had slightly decreased. Moreno et al. also found that through the practice of mindfulness, students learned to improve self-control and self-regulation by studying the effects of the MBI on internalizing and externalizing problems using the “Screening of Emotional Problems and Child Behavior” [43,93]. Finally, one study using the structured mixed-method approach of concept mapping (graphic representation of the perceived changes derived from the ideas generated by participants) found that students stated “I have more self-control with myself” and “I have more self-control with others” after practicing in mindfulness meditation for eight weeks [48]. 

#### 3.4.5. Cognitive Abilities

Concerning cognitive abilities, four studies found improvements after student participation in the MBI [40,41,46]. Two studies measured executive functions (EF) and both used the flanker fish task [51] targeting all three core executive functions [40,41]. Parker et al. found significantly higher EF scores at post-test for the intervention group when compared to the control group [40]. Schonert-Reichl et al. also administered the hearts and flowers version of the dots task in order to assess children’s working memory, response inhibition, and cognitive flexibility [41,51]. Analysis of test scores found no significant difference between the experimental and the control group at baseline. However, the experimental group did show a faster response time in completing each trial compared to the control group. Both studies indicate that the participation of students in MBIs leads to increased inhibitory control, which in turn leads to improved emotional control, decreased aggression, and social problems. Similarly, Kielty et al. did not consider EF effects but reports perceived effects on attentional capacity; teachers reported greater levels of attention and noticeable differences in children with ADHD [49]. 

#### 3.4.6. Social Abilities

Another domain we found in the included studies is the positive effects of school MBIs on social abilities [39,40,41,44,47]. Waldemar et al. used the Strengths and Difficulties Questionnaire—Child Version [55] to assess conduct problems, interpersonal relationship, and prosocial behaviors. Post-intervention results indicated significant improvements in conduct problems, prosocial behaviors, and interpersonal relationships [47]. Parker et al. found significant decreases in social problems and aggression in both boys and girls post-MBI [40]. One study using parent-reported measures of anxiety observed a significant reduction in aggressive behaviors in boys and girls [39]. They also found that children with lower levels of rumination showed a larger increase in the conduct of “attending to others’ emotions” post-intervention, while children with higher rumination at pre-intervention attended more to others’ emotions initially. Teacher reports of social abilities in this study revealed increases in quality of student relationship (student respect, student friendship, sense of belonging), contributing to the shaping of the class environment. Schonert-Reichl et al., used the Interpersonal Reactivity Index [69] adapted for children [41]. Only two of the seven subscales of the questionnaire were used: “empathic concern” (tendency to feel concern for other individuals) and “perspective taking” (tendency to consider things from others viewpoints) in order to examine the effects of MBIs on social awareness and caring for others. Authors also used the 7-item subscale of the Social Goals Questionnaire [73] to measure social responsibilities (keeping promises, showing empathy and respect to other kids). Authors put peer rated measures in place to track pro-sociability and peer acceptance (children’s level of acceptance by peers) [41]. Five types of prosocial behaviors (sharing/cooperation, trustworthiness, helpfulness, kindness, perspective taking/being understanding) and two types of aggressive/antisocial behaviors (rule breaking and fighting) were assessed. Authors found a great decrease in peer-rated aggression, students were rated by peers as more prosocial, and had higher rates in peer acceptance post-intervention [41]. However, Lombas et al. screened empathy levels in students at pre- and post-intervention but the results revealed no effects [44]. 

#### 3.4.7. Mindfulness

Five studies measured the trait mindfulness outcomes of the MBI [40,42,44,49,50]. Kielty et al. used the 15-item Mindful Student Questionnaire [74] to assess three constructs of mindfulness: “receptive attitude”, “attentive awareness”, and “intentionality” [49]. The authors revealed conflicting results, as qualitative comments seemed to indicate an increase in children’s understanding of mindfulness and usefulness over time, while quantitative results showed a decrease in the mindfulness score [49]. Schonert-Reichl et al. opted for The Mindful Attention Awareness Scale adapted for children [58] to study individual differences in the frequency of mindful states over time [41]. The results showed significant improvements in the frequency of mindful states in students from pre- to post-test. Lombas et al.’s results showed that intervention had improved students’ mindfulness competencies, but the effects on mindfulness were dependent on the initial level (pre-test level) [44]. With regard to teachers, Bradley et al. found slight but not significant improvements in teacher mindfulness scores at post-intervention [50]. Similarly, Kuyken et al. found no evidence of differences in mindfulness scores between MBI group teachers and controlled group teachers [42]. At 1-year follow-up, there was only evidence of differences on mindfulness in teaching on an intrapersonal level compared to the control group. 

#### 3.4.8. Environment: School and Class Climates

All studies investigated changes in school environments and found increases in quality of life, improved class or school climates as a result of increased self-control and prosocial behaviors. Two studies focused on MBI teacher outcomes on school and class climates and found increased teacher well-being at post-intervention in relation to higher scores of self-compassion, self-acceptance, contentment, and teaching efficacy [42,50]. Authors found these results had direct positive influences on class climate [50]. Kuyken et al. found significant effects of mindfulness training on teachers as well as after teacher-led MBI [42]. Following teacher mindfulness training, MBI teachers reported better school leadership and involvement than teachers in the control group. After delivering the MBI to students, teachers continued to report a better school climate in terms of leadership, involvement, and in respectfulness. These changes were still accurate in regards to more respectful climate at the 1-year follow-up. In regard to positive school and class climates, all programs contain components that facilitate impacts on safety and community factors (n = 12), seven programs on academic factors, and three on institutional factors (see Table 4). Safety factors were met through MBI programs by: (1) Learning respect; (2) Awareness and regulating emotions and behaviors; (3) Learning ethics and responsibility; (4) Feeling a sense of belonging; (5) Creating a supportive school environment; (6) Improving the sense of well-being. Community factors were met by: (1) Increasing positive and supportive relationships; (2) Social competence; (3) Learning empathy, altruism, compassion, and forgiveness; (4) Perspective taking. The MBIs comprising components with regard to academic factors found that these interventions changed the ecology of the classroom environment, creating a positive classroom environment, and mindful and accepting instruction giving (see Table 4). Finally, regarding the institutional factor of school climate, one study found that mindfulness meditation had positive effects on school functioning [48], one found better school leadership and involvement [42], and one found student positive shaping of the environment; however, this was not further described by the authors [39]. 

## 4. Discussion

This systematic review has examined the developing literature on the effects of mindfulness-based interventions (MBIs) established in schools and the considerations of such interventions for improving youths’ mental health and school climate. The included studies had the common objectives of exploring the feasibility of MBIs in a school context and assessing the effects of such programs on mental health (emotional problems and conduct problems), self-regulation, social–emotional competencies, and quality of life (teacher–student relationship and classroom and school climates) of students in preschool, elementary school, and high school. We had hypothesized that across interventions, the results would be consistent regarding their effects on children and adolescents (decrease in stress, anxiety, and depression) and on improving school and/or class climate. Yet, the results indicate heterogeneity in the published reports in terms of study design, objectives, type of MBI, their implementation, delivery, and assessment. 

First, the MBI programs were theorized and developed based on existing and validated mindfulness programs, such as MBSR and MBCT. However, all of these varied widely in frequency, duration, practices, instructor training, assessments methods, and school and class climate considerations, creating wide discrepancies. Related to mindfulness curriculums, most of these were established by authors for the purpose of their study and/or combined with other types of interventional approaches (SEL, positive psychology, and character strengths), therefore diverging in exercises and practices. For instance, one MBI of the included studies consisted of the practice of meditations (sitting down and in movement), which is a just one of the many mindfulness practices [48]. However, the choice of exercises and practices should strongly be considered and be developmentally appropriate in order to ensure the youth’s adherence to it. If not, the literature suggests that this may counteract the desired benefits by affecting engagement with the MBI or and motivation to complete it [104].

Related to implementation, the included studies greatly differed in their methodology and MBI delivery components; some have methodological limitations that suggest risk of bias, heterogenous sample sizes, lack of active control groups, and a lack of blinding measures. Only five studies applied RCT study designs, only one of which comprised an active control group, eight applied pre-experimental (one-group pre-test-post-test) designs, and one study applied a longitudinal study design. One explanation for this lack of replicability is the implementation limitations that come with school interventions. Indeed, most studies were exploratory in nature, and therefore, had to adapt to school requirements without imposing too many organizational conditions or disturbing the curriculum. Furthermore, research has shown that there is a lack of access to trained mindfulness instructors to provide in-school sessions. To address this issue, teacher trainings in mindfulness allow for qualified teachers to directly implement these mindfulness strategies into their classrooms without disturbing school functioning. This is the case with seven included studies, where teachers became committed educators, applying mindfulness practices within schools. However, this raises instructor training discrepancies and proficiency issues, as there are no set standards or requirements for school MBI trainings for teachers [104,105].

Consistent findings in terms of emotional problems (stress, anxiety, and depression), prosociality, and school climate emerged. A total of ten studies assessed potential mediators of student mental health outcomes, associated with mindfulness competencies and cognitive reactivity, mediating healthy relationships [39,40,41,43,44,45,46,47,48,49]. Specifically, there were positive effects found for behavioral self-regulation (externalized problems), emotional regulation, attentional capacities, executive functioning, ADHD symptoms, and peer and prosocial behavior. Emotional and behavioral regulation was the most frequently studied outcome and was assessed in nine studies. Three of them found improvements in emotional awareness (understanding of emotions, emotional granularity, verbal sharing of emotions, emotional attention, and bodily emotion awareness), and six of them found improvements in emotional and behavioral control, following the MBI. One study found that these results only applied to boys in the intervention group [40], and one study found the levels of emotional control decreased at the 1 year follow-up rather than continuing to improve [46]. Furthermore, seven studies specifically addressed the mental health disorders of depression [39,41,44,47] and anxiety [39,40,50]. The findings of two studies indicate significant decreases in parent-rated anxiety scores (panic disorder, social phobia, generalized anxiety disorder, obsessive compulsive disorder, and separation anxiety disorder) following the MBI [39,40]. Regarding depression, two studies found significant decrease in adolescents’ (mean age 11 years old) depressive symptoms, significant improvements in optimism and in perspective taking at post-intervention [41,47]. Additionally, improved indexes of psychological well-being (relative to self-esteem and satisfaction with life) indicate that MBIs could reduce depressive symptoms in adolescents, such as perceived stress, decreased motivation, increased empathy, life satisfaction, but dependently of mindfulness levels [44]. Nonetheless, only a small portion of the included studies assessed these outcomes, which compromises the generalizability of these observations. Although the current literature has shown the promising effects of MBIs on decreasing depressive symptoms in youth populations [106,107], recent meta-analyses found methodological and implementation variables (sample size and non-active control groups) that compromised the generalizability of these findings [108,109]. In fact, research finds that the type of MBI program (MBCT over MBSR) and longer follow up periods, combined with individual counseling were significant MBI mediators of depression in adolescents [110], which should be considered in future methodological and implementation variables.

Secondly, another aim of the current study was to investigate the effects of MBIs on school and class climates. We had also hypothesized that school MBIs would promote a more positive environment at school, which could benefit students’ and school staff’s well-being. This angle of research was motivated by the inequalities in mental health accessibility. Research showed that school interventions had the potential to increase accessibility to mental health practices, which are usually not made available to certain populations, particularly to low- and middle-income families and countries [111]. Additionally, school climate and teacher–student relationships are usually at risk in these environments and contribute to dissatisfaction with oneself, relationships, and life. Moreover, the existing literature suggests that positive relationships are important for an individual’s health and sense of well-being [112]. Indeed, research found that teachers practicing mindfulness experience enhanced well-being and develop professional resilience and commitment, which positively influences student outcomes [113]. The results seem to be concurrent with previous findings, as the results suggest that MBIs can lead to changes in the school environment by increasing the quality of life for teachers and students. In fact, we found that students’ well-being is a major observed outcome of school-based MBIs, which was improved across intervention types. Regarding positive school climate factors [25], such as safety (emotional safety, discipline, and respect), community (quality of relationships and social skills), academic (quality of curriculums, teacher training, and professional development), and institutional standards, we found that MBIs influenced mostly safety factors. Indeed, results suggest that MBIs could promote emotional safety, order, and respect, as empathy, compassion (kindness, being supportive, friendly attention, and acceptance) and perspective taking were part of each MBI program; they can collectively improve the factors of safety and community. In addition, MBIs with a mixed approach of mindfulness and social emotional learning added teaching the concepts of ethics and responsibility. As a result, students were involved in more positive relationships post-MBI, which could also translate to improved safety and community factors. Additionally, programs delivered by teachers had positive effects on teacher–student relationships due to more mindful and accepting interactions. This further supports an improvement of the community factors as a result of the improved quality of social interactions between school children and teachers, but also of the safety factors affecting students’ sense of emotional safety and relationship to teacher authority. These results suggest that the skills developed throughout school MBIs allow children to have a better understanding of their relationships and an increased respect for peers, including teachers. Generally, we found that school MBIs are not made to address institutional factors, such as structural organization, adequacy, and availability of resources. However, there could be an ad hoc improvement to institutional factors, as shown in three studies, in which teachers reported that students had shaped their environment as a result of the intervention [39], found that school leadership and involvement improved [42], and mindfulness meditation had positive effects on school functioning [48]. 

Overall, the results obtained on school MBIs and their effects on students’ mental health and the environment that contributes to it, such as school climate, are new-found. Therefore, the comparability of the little existing research is compromised as it lacks a general consensus. Thus, this review contributes to the field by suggesting the following implications for future research. First of all, investigating how mindfulness influences factors such as teaching, respect, quality of relationships, academic and institutional functioning could positively influence school climates [25], and help determine the full implications of applying mindfulness practices in an academic context. Future research should clearly define and operationalize school and class climates with the aim of incorporating these considerations into future school-based MBIs. Each study included in this review considered the effects of the MBI on school climate; however, only five were specifically designed with the primary purpose of improving school and class climates. Therefore, it could be beneficial to design a school MBI composed of structured and validated teacher trainings, incorporating specific developmental-stage-appropriate practices and exercises consisting of mindfulness and SEL, adapting the duration and frequency of sessions, all for the holistic objective of improving both student mental health issues and school climate. In addition, future research would benefit from attempting to add to the existing findings by using similar methodologies to those used in existing studies—this implies using the same study design, larger samples, consensus on MBI programs, and assessment tools in order to replicate and validate the findings. This would provide significant insight, as research in this specific field lacks replication. In addition, one of this study’s limitations was the lack of focus on study design factors, such as duration and implementation, during the inclusion criteria selection process, which may have allowed for more comparable search results. Furthermore, some of the current research does look at intervention outcomes for teachers as well as for students; however, if studies are to be successful in investigating MBI effects on school climate, we suggest that using a whole-school design may allow for more insightful data. That is to say that implementing the intervention in a school should also consider the degree of participation when deciding on their participant pool. In view of this, using a whole-school approach can be achieved by having staff and students of every grade involved in the investigation, as well as providing staff relevant training or a professional curriculum to ensure the long-term application and effects of the MBI [114]. Moreover, offering MBIs as standard features of school curriculums, or through a whole-school approach could be viewed as increasing the availability of this resource and would be an improvement towards the institutional factors of the school climate.

## 5. Conclusions

School mindfulness-based interventions have shown their benefits on child and adolescent populations, particularly in emotional and behavioral regulation, reducing stress, anxiety, and depressive symptoms, improving executive functions, and socio-emotional skills. The results of this systematic review also suggest that MBIs could potentially help improve student well-being and environmental factors, such as school and class climates. Specifically, MBI improve the children’s sense of safety and community by improving the quality of their relationships with other students, their peers, and teachers. Thus, offering MBIs as a standard feature of school curriculums, or as a whole-school approach, would increase the availability of this resource and make mental health interventions more accessible to children and adolescents. Nonetheless, these findings should be examined with caution considering the wide discrepancies in terms of types of interventions, instructor trainings, assessment measures, choice of practices and exercises, which make the effects of the existing school MBIs practically impossible to compare. Furthermore, incorporating school climate perspectives, such as whole-school MBI approaches, implies redefining variables of existing models and using replicable and comparable assessment methods, whilst considering the capacities and limitations of the academic and institutional context.

## Figures and Tables

**Table 3:** Included studies outcome measures.

	Implementation and Program Measures	Teacher Assessment Measures	Student Assessment Measures	
			Self-Reported	Teacher or Parent Reported
Parker et al., (2014) [40]	A trained observer rated each teacher’s fidelity of implementation in the three intervention classrooms. Teacher interview for intervention feasibility.		Executive functions: flanker fish task (Diamond et al., 2007) [51]; Intentions to Use Alcohol and Tobacco Scale (Kupersmidt, Scull, & Austin, 2010) [52]	Behavior and emotion regulation: Children’s Behavior Checklist Teachers’ Report Form (C-TRF) [53]; Self-Control Rating Scale (SCRS) [54]
Waldemar et al., (2016) [47]			The Strengths and Difficulties Questionnaire—Child Version (SDQ-C) [55] (hyperactivity, emotional problems, conduct problems, interpersonal relationship, and prosocial behavior); The Youth Quality of Life Instrument (YQOL-R) [56] (personal, relational, environmental, and general); ADHD: The Swanson, Nolan and Pelham Questionnaire–IV (SNAP-IV) [57]	
Lombas et al., (2019) [44]		Teacher Acceptability and Implementation Fidelity Questionnaire	Student Acceptability Questionnaire; Mindfulness (MAAS) [58]; Self-esteem: Rosenberg Self-Esteem Scale [59]; Satisfaction with life: Satisfaction with Life Scale [60]; Depressive symptomatology: Reduced Scale of Depressive Symptomatology [61]; Perceived stress: Perceived Stress Scale [62]; Basic psychological needs: Psychological Needs Satisfaction Scale in Education [63]; Emotional intelligence: Trait Meta-Mood Scale [64]; Empathy: Index of Empathy for Children and Adolescents [65]; School aggression: School Aggression Scale [66]; Academic motivation: Academic motivation according to self-determination theory [67]	Classroom Environment Scale [68]
Schonert-Reichl et al., (2015) [41]	Survey on dosage of implementation		Executive functions: Flanker task and the hearts and flowers version of the dots task [51]; Cortisol salivatory measure; Empathy and perspective: Interpersonal Reactivity Index [69]; Resiliency inventory (RI) (optimism and emotional control subscales) [70]; The school self-concept scale from Marsh’s Self-Description Questionnaire,) [71]; Depression: Seattle Personality Questionnaire for Children [72]; Mindfulness: The Mindful Attention Awareness Scale adapted for children [58]; Social responsibility: subscale of the Social Goals Questionnaire [73]; Peer-nomination for prosociality and peer acceptance	Math grade for achievement measure
Kielty et al., (2017) [49]	Qualitative surveys about experience and curriculum	Teacher Fidelity and Acceptability Measure created for this study.	Mindfulness Student Questionnaire [74]; Positive Experiences at School Scale [75]	
Suárez-García et al., (2020). [46]			The Factor G test (Scale 2—Form A), [76]; Test of Perception of Differences—Revised (CARAS-R) [77]	Evaluation System for Children and Adolescents—school teachers’ version (SENA), subscales: “Attention Problems”, “Hyperactivity-impulsivity”, “Aggression”
Lauren Meyer & Katie Eklund (2020) [45]		The Kentucky Inventory of Mindfulness [78]; My Class Inventory—Teacher Form [79]	Child and Adolescent Mindfulness Measure [80]; My Class Inventory—Short Form Revised [81]	
Wisner, Betsy (2014) [48]	Concept mapping		Concept Mapping and Narrative Questionnaire	
Bradley et al., (2018) [50]	Weekly Teacher Implementation Survey; Biannual Outcome Survey (battery of validated self-report measures)	The Maslach Burnout Inventory Educators Survey [82]; Ryff’s Scales of Psychological Well-Being [83]; The Positive Emotion Assessment of Contentment Scale [84]; The Relationship Satisfaction Scale [85]; The Teachers’ Sense of Efficacy Scale—Short Form [86]; The Self-Compassion Scale—Short Form [87]; The Generic Job Satisfaction [88]; The Perceived Stress Scale 10-Item Inventory [89]; The Cognitive and Affective Mindfulness Scale—Revised 10-item version [90]; Well-Being Survey	Mood Meter Report (emotion plotting tool)	
Kuyken et al., (2022) [42]		The Maslach Burnout Inventory Educators Survey [82]; The Teachers’ Sense of Efficacy Scale [86]; Five-Facet Mindfulness Questionnaire—Short Form [78]; Mindfulness in Teaching Scale [91]; Perceived Stress Scale, PSS [62]; Anxiety and depression (Patient Health Questionnaire); School Climate and Connectedness Survey		
Moreno-Gómez, Luna, & Cejudo (2020) [43]				Behavior Assessment System for Children, second edition [92]; Screening of Emotional Problems and Child Behavior [93]; Average Kindergarten Grade in: (1) Self-knowledge and personal autonomy; (2) Environmental knowledge; (3) Languages: communication and representation.
Van de Weijer-Bergsma et al., (2012) [39]			The Dutch 10-item Non-Productive Thoughts Questionnaire for Children [94]; The Dutch 30-item Emotion Awareness Questionnaire revised [95]; The Dutch Sense of Coherence Questionnaire for Children Subjective Happiness Scale [96]	Dutch Screen for Child Anxiety-Related Emotional Disorders [97]; Social Competence and Behavior Evaluation [98]; Sleep Disturbance Scale for Children [99]; Teacher Report About Class Climate [100]; School as a Caring Community Profile II [101]

**Table 4:** School MBI programs—Part 1.

Study	Waldemar (2016) [47]	Schonert-Reichl (2015) [41]	Parker (2014) [40]	Van de Weijer-Bergsma (2012) [39]	Kielty (2017) [49]	Bradley (2018) [50]
Program	M-SEL		Master Mind	MindfulKids	Author Curricula Based on MindUp and MindfulSchools	The Four Pillars
Theory	M-SEL: Mindfulness- Social–Emotional Learning	Positive Psychology SEL, Mindfulness (MindUp)	Mindfulness (MBSR, MBCT)	Mindfulness (MindfulSchools, MBSR, MBCT)	Mindfulness (MBSR, MBCT)	Positive Psychology, SEL, Mindfulness (MindUp)
Manual	Not available	Available for over 5 years	Available for over 5 years	Not available	Not available	Available less than 5 years
Delivery	Class by non-school trainer (therapist)	Class by non-school trainer and teachers	Class by teachers	Class by non-school trainer/authors	Class by non-school trainer/ authors	Class by teacher
Period/Intensity	12 lessons—1 h long	12 lessons, 1/week 4–50 min, daily 3 min core practice in class	20 lessons, 4-week period, 1 lesson/day 15 min	12 lessons, 6-week period, 2 lessons/week 30 min	3-week period, 30 min lessons, 5 sessions, 1 booster session 1 year later	1-year period, 20 min lesson every other week 15 lessons of Mindfulness, 10 lessons of community, 8 lessons of self-curiosity,10 lessons of contentment
Mindfulness	Mindfulness of breath—reflexive and playful mindfulness activities (mindfulness of eating, fishbowl technique for body awareness, mindful listening, nonjudgment/describing) & CASEL Skills (social emotional)	Breath awareness, psychoeducation, awareness of senses, home practices Kindness practices, group discussion, working on thoughts and emotions	Awareness of the body, breath and sensation, awareness of feelings, thoughts, relationships, Home practice, group discussions, mindful breathing	Bodily awareness, orienting attention, observing sounds and silence, curious attitude, awareness of breath, mindful eating, empathy, awareness of emotions and thoughts, non-judgmental awareness, being nonreactive	Psychoeducation, deep breathing and attention to thoughts, awareness of the body and of emotion	Self-awareness, mindful posture, mindfulness of breath, senses, thoughts, emotions, movement, orienting attention/concentration, self-compassion, psychoeducation
School and Class Climate	Safety: respect, recognizing and managing emotions, ethics, and responsibility. Community: empathy, positive relationships, collaboration.	Safety: belonging, caring. Community: understanding of others, performing acts of kindness for others, collectively engaging in community service learning activities. Academic: changing the ecology of the classroom environment, creating a positive classroom environment.	Safety: self-regulation by awareness, expression and modulation of emotion and behaviors, supportive school environment. Community: perspective taking Academic: Positive teacher–student interactions: mindful and accepting instruction giving.	Safety: respect, belonging. Community: social competence, friendly attention, friendship. Academic: class climate. Institution: student shaping of environment.	Safety: calmness, solving problems. Community: supportive relationships.	Safety: calmness, peacefulness well-being. Community: learning prosocial behaviors, altruism, empathy, compassion, forgiveness, taking perspective, self-acceptance.
School MBI programs—Part 2.						
Study	Wisner (2014) [48]	Kuyken (2022) [42]	Moreno (2020) [43]	Suarez-Garcia (2020) [46]	Lombas (2019) [44]	Meyer (2020) [45]
Program	Mindfulness Meditation (MM)	School-Based Mindfulness Training (SBMT)	Mindkinder	Mindkeys Training	Happy Classrooms Program (HCP)	Mindful Moments Intervention
Theorization	MBSR	Author curricula based on MBCT-L	Author curricula based on Bakosh et al. (2015), Kabat-Zinn (2003), Gueldner & Feuerborn (2016), Carsley (2015), Poehlmann-Tynan et al. (2016)	Author curricula based on mindfulness practices and activities	Mindfulness and character strengths practices (Arguis et al. 2012)	Author curricula based on MBSR Kabat-Zinn (2003)
Manual	Not available	Not available	Not available	Not available	Available less than 5 years	Available than 5 years
Delivery	Class by mindfulness expert	Class by teachers after training	Class by a kindergarten teacher after training + assistance of an external instructor	Mindfulness daily activities by teacher after training + mindfulness expert	Class by a teacher after training	Class by teacher after training
Period/Intensity	8 weeks, two to four times a week, 4-to-10 min activities	10 lessons, 30 to 50 min each, over one school term	6 months 6 weekly sessions of 15 min, same time and place each week. Beginning of the classes in different shifts. (4 weeks—12 h training course for teachers on mindfulness techniques)	8 weeks, 1 h, once a week	18 weeks, approximately 5 min, minimum periodicity of twice a week	10-week 2 min mindfulness-based intervention 3 times a day
Mindfulness	Meditation in a stable posture; students were asked to observe the breath and to observe sensations in the body	Combination of psychoeducation, class discussion, and brief mindfulness practices. The program includes suggested home-based mindfulness practices at the end of each session, which are reviewed at the start of the next session	Audio-guided meditations; visualizations, using students’ imagination and their ability to abstract; visualization development of pedagogical and concentration dynamics using mandalas; corporal expression: students’ body awareness activities	Read and debate about mindfulness; breathing techniques (focusing attention); conscious attention to an activity; cultivating kindness and gratitude. (1) Sounding a singing bowl and silence; (2) Explanation of a breathing technique; (3) An attention to sound exercise; (4) Reading a story and a debate; (5) Week’s challenge; and (6) Sounding the singing bowl	Adapted meditation practices of focusing and monitoring attention, mindful movement, breathing exercises, mindful walking, body scan, mindful eating. + character strengths and well-being practices: development of appreciation of beauty, gratitude, hope, humor, and spirituality	19 mindfulness-based movement, breathing, stretching, and body awareness exercises
School/ Class Climate	Safety: perceived enhancements in emotional coping reflect intrapersonal and psychosocial benefits. Calmer and more peaceful school climate with enhanced student engagement. Community: Accepting and supportive environment. Meditation helped teachers change their moods and improve stress management. Institution: perceived school climate benefits of meditation with regard to their school functioning.	Safety: better student engagement (self-efficacy) Community: teachers reported a respectful school climate, post-intervention and at 1-year follow up. Institution: better school leadership and involvement.	Safety: significant improvement in some indicators of school behavioral problems. Focusing on intrapersonal skills, such as the recognition of emotions, emotional and behavioral self-regulation, promotion of emotional well-being. Community: significant improvement in school adaptation.	Safety: Teacher and student well-being improvement, mainly through improvements in their capacity for emotional regulation and a reduction in stress. Community: Teacher stress reduction positively affects the relationship with students and classroom relationship management, contributing to an improved model of behavior for students. Academic: Available teacher training and resources of mindfulness for professional development.	Safety: potential reduction in school aggression, physical and relational. Community: improvements in relatedness. Academic: increases in factors relating to academic motivation.	Safety: higher ratings of satisfaction at post-intervention. Community: lower scores reported for friction and competitiveness, changes in reported friction and cohesion post-MBI.

## Data Availability

Not applicable.
